# Dietary Synbiotics Can Help Relieve the Impacts of Deltamethrin Toxicity of Nile Tilapia Reared at Low Temperatures

**DOI:** 10.3390/ani11061790

**Published:** 2021-06-15

**Authors:** Mahmoud S. Gewaily, Safaa E. Abdo, Eman M. Moustafa, Marwa F. AbdEl-kader, Ibrahim M. Abd El-Razek, Mohamed El-Sharnouby, Mohamed Alkafafy, Sayed Haidar Abbas Raza, Mohammed F. El Basuini, Hien Van Doan, Mahmoud A. O. Dawood

**Affiliations:** 1 Department of Anatomy and Embryology, Faculty of Veterinary Medicine, Kafrelsheikh University, Kafr El Sheikh 33516, Egypt; drmahmoud_gewaily@yahoo.com; 2 Department of Animal Wealth Development, Faculty of Veterinary Medicine, Kafrelsheikh University, Kafr El Sheikh 33516, Egypt; safaa_2m@yahoo.com; 3 Department of Fish Diseases and Management, Faculty of Veterinary Medicine Kafrelsheikh University, Kafr El Sheikh 33516, Egypt; emantarek2002@yahoo.com; 4 Department of Fish Diseases and Management, Sakha Aquaculture Research Unit, Central Laboratory for Aquaculture Research, A.R.C., Kafr El Sheikh 33516, Egypt; marwa.abdelkader@vet.kfs.edu.eg; 5 Department of Animal Production, Faculty of Agriculture, Kafrelsheikh University, Kafr El Sheikh 33516, Egypt; ibrahim.abdelrazek1@agr.kfs.edu.eg (I.M.A.E.-R.); mahmouddawood55@gmail.com (M.A.O.D.); 6 Department of Biotechnology, College of Science, Taif University, P.O. Box 11099, Taif 21944, Saudi Arabia; m.sharnouby@Tu.edu.sa (M.E.-S.); m.kafafy@tu.edu.sa (M.A.); 7 State Key Laboratory of Animal Genetics Breeding & Reproduction, College of Animal Science and Technology, Northwest A&F University, Yangling 712100, China; dr.haiderabbasraza@gmail.com; 8 Faculty of Desert Agriculture, King Salman International University, South Sinai 46618, Egypt; m_fouad_islam@yahoo.com; 9 Department of Animal Production, Faculty of Agriculture, Tanta University, Tanta 31527, Egypt; 10 Department of Animal and Aquatic Sciences, Faculty of Agriculture, Chiang Mai University, Chiang Mai 50200, Thailand; 11 Science and Technology Research Institute, Chiang Mai University, 239 Huay Keaw Rd., Suthep, Muang, Chiang Mai 50200, Thailand

**Keywords:** deltamethrin, synbiotic, Nile tilapia, histopathology, inflammation, suboptimal temperature

## Abstract

**Simple Summary:**

The toxic impacts of pesticides and insecticides are strongly correlated with water temperature. Water temperature can increase or decrease the efficacy of toxins and their influence on aquatic organisms. An alternate approach to augmenting fish resistance to ambient deltamethrin (DMT) toxicity and low water temperature via synbiotic feeding was proposed. In this study, fish were allocated into four groups and kept at suboptimal water temperature (21 ± 2 °C): control, DMT, synbiotic, and DMT + synbiotic. The results illustrate that including synbiotics in the Nile tilapia diet stimulates the immunity and antioxidant systems in fish, enabling the fish reared at a suboptimal temperature to counteract the immunity suppression and oxidative stress caused by DMT exposure.

**Abstract:**

The optimal water temperature for the normal growth of Nile tilapia is between 26 and 28 °C, and the toxicity of pesticides is strongly related to water temperature. An alternate approach to augmenting the resistance of fish to ambient water toxicity and low water temperature via synbiotic feeding was proposed. In this study, fish were allocated into four groups with 10 fish in each replicate, where they were fed a basal diet or synbiotics (550 mg/kg) and kept at a suboptimal water temperature (21 ± 2 °C). The prepared diets were fed to Nile tilapia for 30 days with or without deltamethrin (DMT) ambient exposure (15 μg/L). The groups were named control (basal diet without DMT toxicity), DMT (basal diet with DMT toxicity), synbiotic (synbiotics without DMT toxicity), and DMT + synbiotic (synbiotics with DMT toxicity). The results displayed upregulated transcription of catalase, glutathione peroxidase, and interferon (IFN-γ) genes caused by DMT exposure and synbiotic feeding when compared with the controls. Moreover, HSP70 and CASP3 genes displayed increased transcription caused by DMT exposure without synbiotic feeding. However, fish fed with synbiotics showed downregulated HSP70 and CASP3 gene expressions. The transcription of IL-1β and IL-8 genes were also decreased by DMT exposure, while fish fed synbiotics showed upregulated levels. DMT exposure resulted in irregular histopathological features in gills, intestine, spleen, and liver tissues, while fish fed synbiotics showed regular, normal, and protected histopathological images. Our results indicated that dietary synbiotics ameliorated histopathological damages in DMT-exposed tilapia through alleviation of oxidative stress and inflammation as well as enhancing the immunity.

## 1. Introduction

Aquatic pollutants constitute a significant problem that threatens the basic requirements for aquaculture-derived food [1]. The shortage of water resources has forced fish farmers to reuse agricultural drainage water, which might contain pesticides and insecticides [2]. The continuous exposure to toxic derivatives results in oxidative stress, thereby causing immunosuppression and a high possibility of infection attacks [3,4]. Several studies clarified the negative impact of pesticides and insecticides on the production of finfish species and their health status. Traditionally, deltamethrin (DMT) is applied as a model pesticide in the agriculture sector, and it can be present in refluxed agricultural drainage water, leading to harmful impacts on the ecological system [5]. High levels of DMT derivatives induce oxidative stress and systemic and mucosal inflammation in finfish species [6]. In this regard, the immune cells and functional cells lose their function to protect fish from stressors and infection [7,8].

The identification of environmentally friendly alternative substances that can reduce the usage of antibiotics in aquaculture is highly recommended [9,10,11]. Natural functional supplements are substantial factors with high potential to enhance aquatic organisms’ antioxidative and immune responses [12,13]. Probiotics, prebiotics, their mixture, i.e., “synbiotics”, and natural feed ingredients such as insect meal have been shown to be applicable supplements in aquafeed with immunostimulant ability [14,15,16]. Indeed, synbiotics boast the combined effects of both pro- and prebiotic supplements, beginning from the activation of the local intestinal immunity and the related entire body immunity [17]. Many studies investigated the effects of synbiotics as functional growth enhancers, immunostimulant agents [18,19], and antioxidative factors [20,21]. Moreover, synbiotics were validated as anti-inflammatory agents with a high capacity to decrease the impact of stressors [22,23] on the performance of finfish species [14]. Heat-killed beneficial bacterial cells, “paraprobiotics”, are a new form of probiotics and were introduced to the aquafeed industry, as they can potentiate aquatic animals’ growth performance, immunity, and well-being [24,25]. In this context, dietary-inactivated *Lactobacillus plantarum* L-137 cells (LP20) were successfully included in aquafeed and resulted in improved growth behavior, digestibility, and health conditions for several aquatic animals [26,27,28]. Yeast cell-derived substances, such as β-glucan, were also demonstrated as functional immunostimulants when included in aquafeed [29]. The mixture of LP20 and β-glucan was investigated in several studies and approved as an active immunobiotic in aquaculture [30,31,32,33]. In our previous study, a dietary mixture of LP20 and β-glucan enhanced the growth performance, hematobiochemical indices, and immune response of Nile tilapia. Concurrently, Nile tilapia treated with a mixture of LP20 and β-glucan displayed high resistance against DMT toxicity [30]. Nevertheless, the present study tested the influence of the dietary LP20 and β-glucan mixture on the histopathological features, antioxidant status, and anti-inflammation induced by DMT in Nile tilapia. 

Nile tilapia is known globally as a feasible commercial fish species with high tolerance to environmental stressors [34]. The optimal growth performance of Nile tilapia requires a stable water temperature between 26 and 28 °C, while higher temperatures and suboptimal water temperature affect the regular performance of the fish [35]. In Egypt, the water temperature decreases below the optimal level during wintertime. Under low-temperature conditions, fish suffer from low feed consumption due to their reduced metabolism [36]. The toxic impacts of pesticides and insecticides are strongly correlated with water temperature, as it can increase or decrease the efficacy of toxins and their influence on aquatic organisms [37]. In this regard, Dawood et al. [30] reported that dietary synbiotics alleviated the negative impacts on the growth performance, blood health, and immune response of Nile tilapia. In our previous study, we examined the impact of DMT toxicity on the growth performance indices of Nile tilapia fed dietary synbiotics [30]. Herein, this research aimed to evaluate the protective effects of synbiotic inclusion on the transcription of immune genes, antioxidant capacity, and pro-inflammatory cytokine levels in the liver as well as histopathological impacts related to inflammation of Nile tilapia under DMT exposure.

## 2. Materials and Methods

### 2.1. Fish, Diets, and Experimental Design

Two sets of diets were formulated by supplementing the basal diet with 0 or 550 mg synbiotic/kg (500 g β-glucan Daigon do, Tokyo, Japan + 50 mg of heat killed *Lactobacillus plantarum*, 2 × 10^11^ CFU per g (LP20), House Wellness Foods Corp., Itami, Japan) [38]. The formulation of the basal diet was previously described by Gewaily et al. [39] and Dawood et al. [40]. To prepare the test diets, fish meal, soybean meal, wheat bran, yellow corn, gluten, starch, dicalcium phosphate, vitamin, and mineral mixture ingredients were mixed; then, 30–40% water was added. The synbiotic mixture (550 mg/kg diet) was mixed with fish oil and added to the basal diet ingredients. All ingredients, synbiotic additives, fish oil, and water were mixed and pelleted using a meat mincer to produce a dough with a 1 to 2 mm die. Prepared pellets were air dried for 24 h and stored in a dry place. The formulated diets were analyzed using the standard method [41]. Table 1 shows the formulation and nutrient composition of the test diets. The prepared diets were fed to Nile tilapia with or without DMT ambient exposure (15 μg/L) (98.5%, Kafr El-Zayat Company for Chemicals and Pesticides, El-Gharbeya, Egypt) for 30 days. The doses of the synbiotic mixture and DMT were proposed by following the methods of Dawood et al. [30] and Cengiz et al. [42], respectively. 

**Figure 1:**
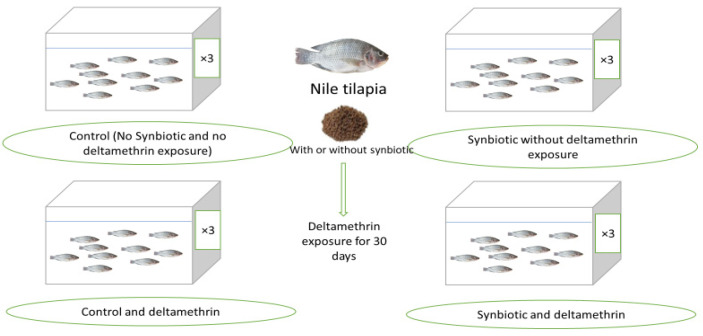
Schematic summary of the study protocol.

Fish were collected from a local farm and transferred to Sakha Aquaculture Research Unit, Kafrelsheikh, Egypt. After acclimatization for 1 week (with basal diet), Nile tilapia (28.21 ± 1.34 g) were randomly allocated to 12 glass aquaria (60 L). Each experimental aquarium was provided with a continuous electric aerator, and half of the water in each tank was exchanged daily with freshly dechlorinated water. Then, fish were allocated to four groups (triplicates) with 10 fish in each replicate, where they were fed the basal diet or synbiotics (550 mg/kg basal diet) and kept at a suboptimal water temperature (21 ± 2 °C). The prepared diets were fed to Nile tilapia with or without DMT ambient exposure (15 μg/L). Fish were fed the test diets by hand for 30 days twice daily (08:00 and 15:00) at 3% of the bodyweight. The groups were named control (basal diet without DMT toxicity), DMT (basal diet with DMT toxicity), synbiotic (synbiotic without DMT toxicity), and DMT + synbiotic (synbiotic with DMT toxicity) (Figure 1).

DMT was added to the aquaria daily to keep the final concentration fixed at 15 μg DMT/L. Fish were fed with test diets up to the satiation level 2 times (08:00 and 16:00) daily. The farming environment was under a natural day and night cycle (12:12 h). The water quality in each aquarium was checked weekly and reported. The water temperature was 21 ± 2 °C, with pH 7.1 ± 0.8, dissolved oxygen 6.5 ± 0.5 mg/L, and total ammonia 0.23 ± 0.03 mg/L.

### 2.2. Histopathology Study

The histopathological study was carried out by following the method of Gewaily et al. [39], where three fish per aquarium (N = 9) were collected, and their viscera were dissected. Then, the intestines, livers, spleens, and gills were separated and fixed in Bouin’s solution for 18–24 h. The tissues were dehydrated using alcohol, cleared in xylene, and embedded in paraffin wax [43]. Then, 5 μm thick sections were obtained with a rotatory microtome (RM 20352035; Leica Microsystems, Wetzlar, Germany) and stained with hematoxylin and eosin stain. Finally, the stained tissue sections were viewed and imaged with a digital camera connected to a BX50/BXFLA microscope (Olympus, Tokyo, Japan).

### 2.3. Transcriptome Assay

Three fish from each aquarium (N = 9) were selected for liver dissection at the end of the trial and frozen at −80 °C for RNA extraction. Fifty milligrams of the liver was used to extract RNA using Trizol (iNtRON Biotechnology, Inc., Gyeonggi-do, Korea) following the manufacturer’s guidelines. The quantity and quality of RNA were checked with a NanoDrop (UV–Vis spectrophotometer Q5000/ Quawell, San Jose, CA, USA). The preparation of cDNA was carried out using a SensiFAST™ cDNA synthesis kit (Bioline, London, UK) following the manufacturer’s guidelines. The primers of heat shock protein 70 (HSP70) [44], caspase-3 (CASP3) [45], catalase (CAT) [46], glutathione peroxidase (GPx) [47], interleukin 1β (IL-1β) [48], interleukin 8 (IL-8) [49], and interferon-gamma (IFN-γ) [48] genes were designed by following the method of Gewaily et al. [39]. Real-time PCR (Stratagene MX3000P) was applied for gene expression using the SYBR Green method (Sensi-Fast SYBR Lo-Rox kit, Bioline, London, UK. The mixture contained 20 μL of 10 μL SYBR mastermix + 0.5 μM of each primer + 2 μL cDNA. The reaction conditions were 10 min at 95 °C, followed by 40 cycles of 15 s at 95 °C, 30 min at 60 °C, and finally 5 min at 85 °C (except IFN-γ, which was at 61 °C) for 1 min. For each mRNA, gene expression was corrected by the β-actin content as a housekeeping gene [49]. The gene expression data were calculated by following the method of Livak and Schmittgen [50].

### 2.4. Statistical Analysis

Levene’s test examined variance homogeneity of data to confirm the normality and homogeneity of data. If the variance homogeneity threshold could be met, the data were analyzed with Duncan’s test. All data were analyzed using one-way analysis of variance (ANOVA) with SPSS 22.0 software (version 22, SPSS Inc., Armonk, NY, USA) and are shown as means ± standard deviation (SD) at *p* < 0.05. 

## 3. Results

### 3.1. Histopathological Image

**Figure 2:**
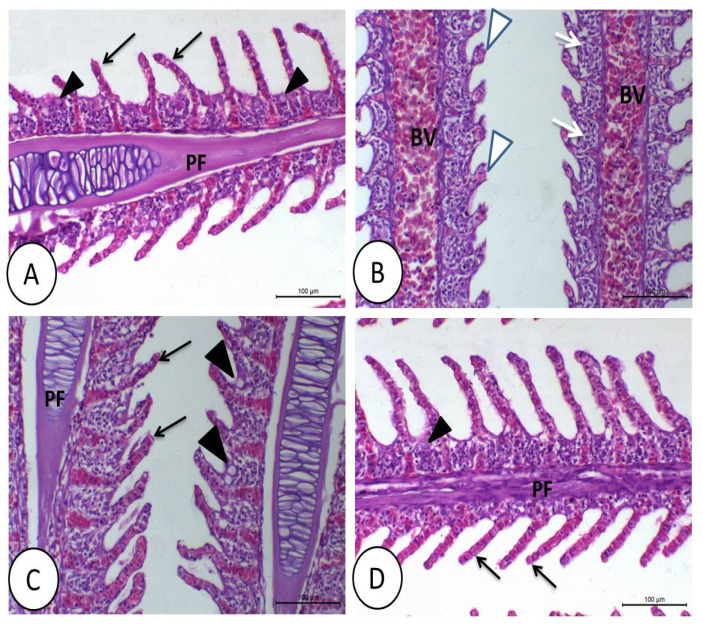
Histology of gills of Nile tilapia in the control (**A**), deltamethrin (DMT) (**B**), synbiotic (**C**), and DMT with synbiotic (**D**) groups. In (**A**,**C**,**D**), the gills show normal histological structures, including primary filaments (PF), secondary filaments (black arrow), and mucous cells (black arrowhead) between the secondary filaments. The toxic effect of DMT (**B**) causes telangiectasia and erosion of secondary filaments (white arrowhead), congestion of blood vessels of primary filaments (BV), and degeneration of epithelial lining (white arrow). H&E staining; bar = 100 µm.

The gills showed congested and large blood vessels in the primary filaments caused by DMT exposure. The apical ends of secondary filaments were dilated, with the erosion of cells in some areas (Figure 2B). However, the gills of the tilapia fed control or synbiotics showed a healthy histological structure (Figure 2A,C,D).

**Figure 3:**
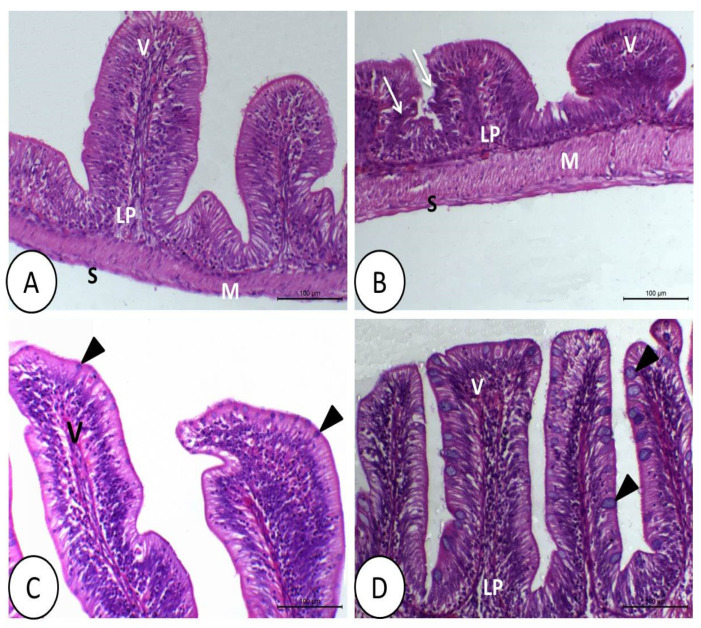
Histology of intestine of Nile tilapia in the control (**A**), deltamethrin (DMT) (**B**), synbiotic (**C**), and DMT with synbiotic (**D**) groups. In (**A**), the intestine shows normal histological structures, including the intestinal villi (V), lamina propria sub mucosa (LP), tunica muscularis (M), and tunica serosa (S). The toxic effect of deltamethrin (**B**) decreases the number of intestinal villi with degeneration of the epithelial lining (white arrow) and leukocytic infiltration. In (**C**,**D**), the intestine has a normal appearance like that in the control group. The intestinal villi increase in number, height, and width with prominent goblet cells (black arrowhead) and without a toxic effect of deltamethrin in group (**D**). H&E staining; bar = 100 µm.

The intestine of tilapia fed control or symbiotic diets showed a healthy structure (Figure 3A,C). DMT impeded the growth of the intestinal villi, with some collapse and destruction of the cell lining (Figure 3B). However, in fish fed synbiotics only, the intestinal epithelium revealed a very clear, simple columnar epithelium with many goblet cells. The intestinal villi were characterized by increased thickness and height (Figure 3C). However, fish that were fed with synbiotics and exposed to DMT showed not only a normal epithelium but also increased number, width, and height of the villi, as well as many prominent goblet cells (Figure 3D).

**Figure 4:**
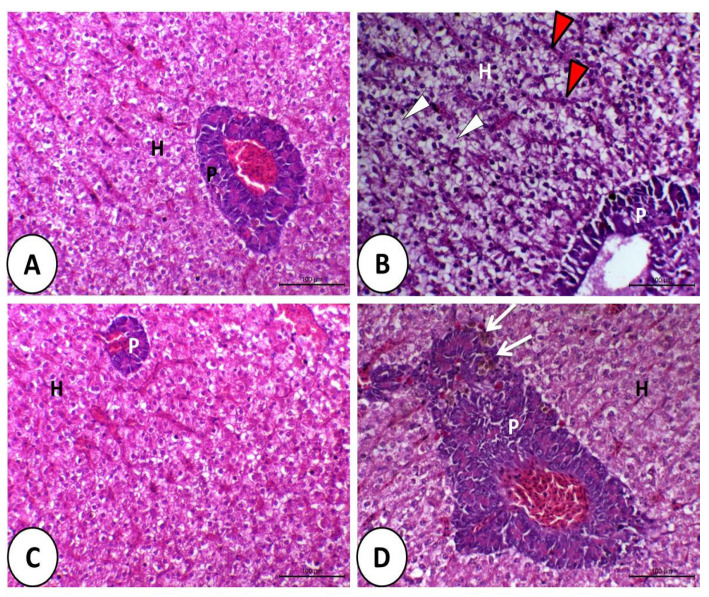
Histology of liver of Nile tilapia in the control (**A**), deltamethrin (DMT) (**B**), synbiotic (**C**), and DMT with synbiotic (**D**) groups. In A and C, the hepatopancreas consists of polyhedral hepatocyte (H) and pancreatic cells (P). The toxic effect of deltamethrin (**B**) causes fatty degeneration (white arrowhead) of hepatocytes and congestion of blood sinusoids (red arrowhead). In (**D**), the hepatopancreas has a relatively normal structure in addition to some melanomacrophages (white arrow), especially in the pancreatic part (P). H&E staining; bar = 100 µm.

The liver of fish fed control or synbiotic diets without DMT exposition appeared normal (Figure 4A,C). The liver in the DMT group was vacuolated, and most of the hepatocytes showed fatty erosion and pyknotic nuclei with congested and dilated blood sinusoids (Figure 4B). By feeding synbiotics, the liver retained its normal structure in fish exposed to DMT (Figure 4D).

**Figure 5:**
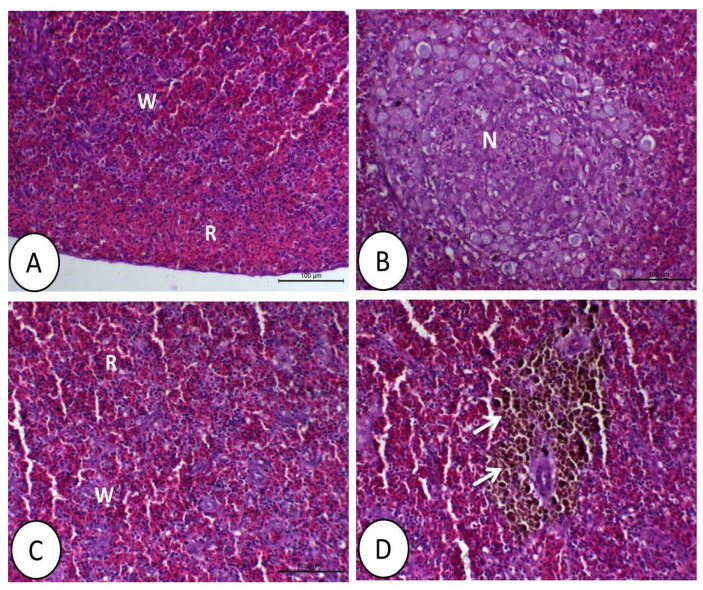
Histology of spleen of Nile tilapia in the control (**A**), deltamethrin (DMT) (**B**), synbiotic (**C**), and both DMT with synbiotic (**D**) groups. In A and C, the spleen consists of white (W) and red pulps (R) that increase in group (**C**). In the deltamethrin group (**B**), the splenic tissue reveals a large area of necrosis (N). In (**D**), the splenic tissue has a relatively normal structure with increased melanomacrophages (white arrow). H&E staining; bar = 100 µm.

The control and synbiotic groups showed a normal structure of the spleen (Figure 5A,C). However, in the DMT group, there was a large area of necrosis with decreased white bulbs compared with the control and synbiotic groups (Figure 5B). There was no sign of necrosis in the spleen in fish fed synbiotics and exposed to DMT. Moreover, melanomacrophages were aggregated around the blood vessels of the splenic tissue (Figure 5D).

### 3.2. Gene Transcription

**Figure 6:**
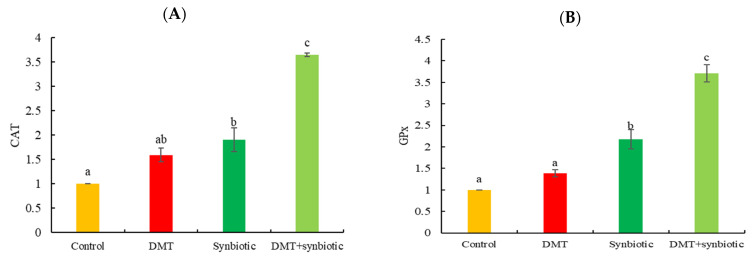
Transcription of antioxidative genes: (**A**) catalase (CAT) and (**B**) glutathione peroxidase (GPx) in Nile tilapia treated with deltamethrin (DMT) with synbiotic feeding. Bars represent mean ± SD (n = 3), and different letters show significant differences (*p* < 0.05).

Fish fed synbiotics and exposed to DMT displayed increased transcription of CAT and GPx genes (*p* < 0.05; Figure 6A,B). A similar trend was observed in fish fed synbiotics without DMT exposure (*p* < 0.05).

**Figure 7:**
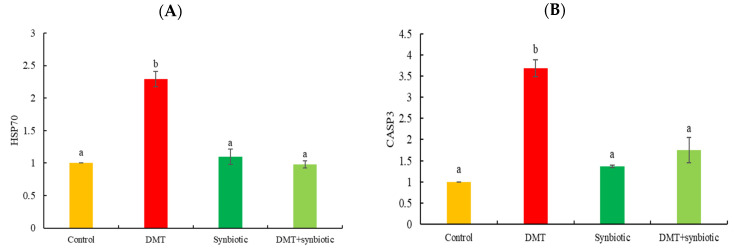
Transcription of (**A**) heat shock protein 70 (HSP70) and (**B**) caspase 3 (CASP3) in Nile tilapia treated with deltamethrin (DMT) with synbiotic feeding. Bars represent mean ± SD (n = 3), and different letters show significant differences (*p* < 0.05).

HSP70 and CASP3 genes exhibited increased transcription in fish exposed to DMT in the absence of synbiotic feeding (*p* < 0.05; Figure 7A,B).

**Figure 8:**
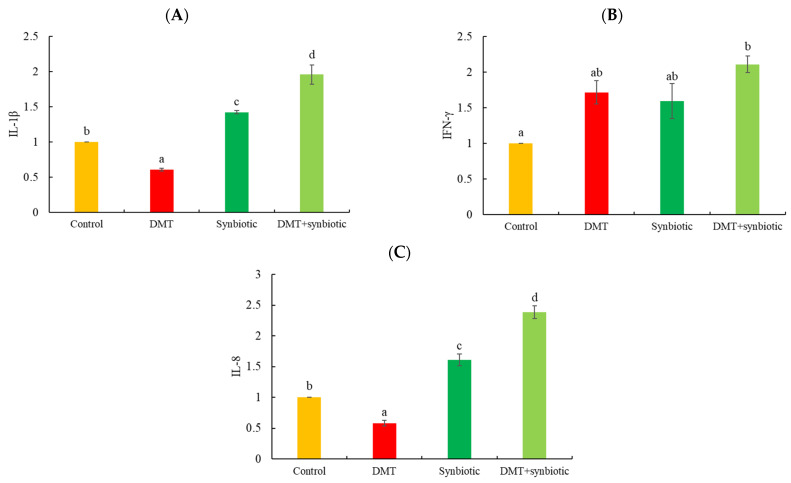
Transcription of (**A**) interleukin 1β (IL-1β), (**B**) interferon-gamma (IFN-γ), (**C**) interleukin 8 (IL-8) in Nile tilapia treated with deltamethrin (DMT) with synbiotic feeding. Bars represent mean ± SD (n = 3), and different letters show significant differences (*p* < 0.05).

IL-1β was downregulated in tilapia with DMT exposure and upregulated by synbiotic and DMT + synbiotic (*p* < 0.05; Figure 8A). However, compared to the control, IFN-γ displayed significantly increased transcription when fish were exposed to DMT and fed synbiotic (DMT + synbiotic) (*p* < 0.05; Figure 8B). DMT resulted in lower IL-8 transcription than that in the other groups (*p* < 0.05; Figure 8C). Fish fed synbiotics and exposed to DMT (DMT + synbiotic) showed more IL-8 upregulation than that in the other groups (*p* < 0.05). Notably, fish exposed to DMT without symbiotic feeding showed the lowest expression of IL-8 (*p* < 0.05).

## 4. Discussion

The ecosystem comprises numerous environmental stressors involved in various biological responses [51,52]. High water temperature usually leads to high absorption rates of pesticides compared to low temperatures [53]. However, continuous exposure to pesticides can also harm aquatic organisms’ performance and health, regardless of the water temperature [54,55]. The current trial was conducted under a suboptimal water temperature (21 ± 2 °C). Normally, Nile tilapia can consume food and grow well under temperatures ranging between 25 and 28 °C [35], but low temperature weakens the growth and activity of fish [56]. In this sense, the accumulation of DMT in the rearing water of Nile tilapia suffering from abnormal feeding habits may lead to suppressed immune and antioxidative responses resulting from inflammation in different body tissues. It has been reported that ambient DMT exposure can harm aquatic animals through impairing the physiological, immunological, and pro-inflammatory responses [11], which has been further confirmed in the present study. Synbiotic application is one of the most effective tools that has been recently reported in aquafeed [18,19]. Synbiotics are a mixture of probiotics and prebiotics, and they can effectively accelerate the resistance of fish against stress through the role of prebiotics in providing the beneficial bacteria (probiotics) with the energy and nutrients, thereby demonstrating their immunomodulation effects [57]. Furthermore, prebiotics themselves have immunomodulation and antioxidative roles [58]. Thus, it has been hypothesized that including synbiotics in tilapia diets may relieve the severe impacts of DMT exposure in rearing water.

The combined exposure to multiple stressors causes DNA damage during cell division [37]. Concurrently, the histological features of gills, intestines, livers, and spleens of tilapia are expected to deteriorate and lead to histopathological inflammation that suppresses immunity and antioxidative conditions [59]. Furthermore, the multiple stressors induce oxidative stress and allow the generation of free radicals, which damage the DNA and tissues [51]. However, synbiotic feeding helps in protecting fish intestines, spleens, and liver tissues from DMT-induced stress.

Tumor necrosis factor-α (TNF-α) and interleukin 8 (IL-8) are pro-inflammatory molecules functioning as inflammation regulation factors [60]. The overproduction of reactive oxygen metabolites (ROS) is the leading cause of oxidative stress, resulting in loss of cell function [61]. High ROS levels break down the lipids, causing lipid peroxidation that can induce cellular oxidative damage [62]. Under high oxidative damage, cells secrete antioxidative enzymes to degenerate the overproduced ROS and maintain the antioxidation balance [63]. Similarly, in the current study, we observed that DMT exposure decreased CAT and GPx gene transcription, while synbiotic feeding increased CAT and GPx gene expressions. Synbiotics as feed additives have been widely used in several fish species [20,21]. Interestingly, high antioxidation capacity against DMT toxicity is probably related to synbiotics as a functional antioxidation agent.

In the present study, the inclusion of synbiotics in the diet of tilapia increased their tolerance to DMT toxicity by increasing the antioxidative and anti-inflammatory responses due to the presence of peptidoglycan and lipopolysaccharides [4,64,65]. El-Murr et al. [4] and Dawood et al. [6] assumed that using β-glucan or *L. plantarum* markedly led to high antioxidation and immunity to cope with the impacts of fipronil or DMT in Nile tilapia. More recently, Nile tilapia fed probiotics, prebiotics, and synbiotics displayed enhanced hemato-immunological responses under DMT toxicity [30]; however, the present study presents a deep interpretation via transcriptomic and histopathological tools.

The upregulation of IFN-γ, IL-8, and IL-1β genes suggested increased resistance against stressors. In this sense, the results confirmed the protective role of synbiotics against inflammation and immunosuppression induced by DMT and low temperature via activating the IFN-γ, IL-8, and IL-1β factors. Synbiotics are speculated as functional additives with the ability to stimulate T lymphocytes [4,64,65]. 

The environmental stressors are the main reason for the high expression of HSP70 involved in alleviating apoptosis in the cells [66,67,68]. The results showed upregulated HSP70 in fish exposed to DMT toxicity, but dietary synbiotics lowered HSP70 expression. The results illustrated that the tested synbiotic is associated with antistress efficacy in fish.

CASP3 is also involved in apoptosis and is responsible for cellular DNA fragmentation during stress [45,69]. The results showed upregulated CASP3 in fish with DMT-induced stress but downregulation in the case of dietary synbiotics, indicating the protective role of synbiotics against DMT-induced apoptosis in Nile tilapia.

The above results clearly show the depressed immunity, antioxidative, and anti-inflammatory responses of Nile tilapia exposed to DMT toxicity under a suboptimal water temperature. However, the dietary synbiotic mixture alleviated the inflammation and oxidative stress induced by DMT and low water temperature. The exact mode of action of synbiotic efficacy can be explained by its immunomodulation activity [70,71], which enhances the local intestinal immunity and protects intestinal barriers from the expected toxicity induced by DMT in the rearing water. More specifically, signals related to immunity show correlations between the local intestinal immunity and the innate immune cells in the fish body [72]. Beneficial microbial cells and glucans can also be easily accessed through specific receptors on the immune cells to enhance cell immunity [73]. The enhanced antioxidative status can be attributed to the synbiotics’ role in activating immunity under the current trial conditions. Additionally, it is suggested that the synbiotic mixture could increase the feed intake of fish regardless of the low water temperature, which would lead to more available nutrients required to enhance the metabolic functions related to deterioration induced by DMT in the different body organs. In this regard, synbiotics are known for their growth-promoting and metabolic regulation activities, as described previously in several studies [74,75]. Thus, it is recommended that future studies reveal the potential roles of synbiotics in the feed utilization of finfish species reared at suboptimal water temperatures.

## 5. Conclusions

In conclusion, including synbiotics in the diet of Nile tilapia stimulates the immunity and antioxidant system in the fish, which enables the fish reared at a suboptimal temperature to counteract the immunity suppression and oxidative stress caused by DMT exposure. Furthermore, fish fed synbiotics showed regular, healthy, and protected histopathological images. 

## Figures and Tables

**Table 1:** Basal diet formulation and chemical composition.

Ingredient	(%)	Composition	(%)
Fish meal	8	Dry matter	90.66
Soybean meal	42	Crude protein	30.05
Wheat bran	10	Ether extract	6.22
Yellow corn	20	Crude fibers	4.95
Gluten	6	Total ash	3.95
Fish oil	3	Gross energy (KJ/g) *	18.98
Dicalcium phosphate	1		
Vitamin and mineral mixture	2		
Vitamin C	0.08		
Starch	7.92		

* Gross energy was calculated based on the values for protein, lipid, and carbohydrate as 23.6, 39.5, and 17.2 kJ/g, respectively.

## Data Availability

The datasets generated during and/or analysed during the current study are available from the corresponding author on reasonable request.
